# Acceptability of a Sublingual Drug Formulation for Respiratory Tract Infections in Children Aged 3 to 5 Years

**DOI:** 10.3390/pharmaceutics13020294

**Published:** 2021-02-23

**Authors:** Andrzej Emeryk, Thibault Vallet, Ewelina Wawryk-Gawda, Arkadiusz Jędrzejewski, Frederic Durmont, Fabrice Ruiz

**Affiliations:** 1 Department of Lung Diseases and Rheumatology in Children, Medical University in Lublin, Aleje Racławickie 1, 20-059 Lublin, Poland; a.emeryk@umlub.pl (A.E.); ewelina.wawryk@wp.pl (E.W.-G.); 2 Alergotest s.c. Specjalistyczne Centrum Medyczne, Jana Sapiehy 2/Lok. 4A, 20-095 Lublin, Poland; arkadiusz.jedrzejewski@umlub.pl; 3 ClinSearch, 110 Avenue Pierre Brossolette, 92240 Malakoff, France; thibault.vallet@clinsearch.net; 4 Department of Pediatric Nursing, Medical University in Lublin, Aleje Racławickie 1, 20-059 Lublin, Poland; 5 Lallemand Pharma AG, Via Selva 2, C.P 5183, 6900 Massagno, Switzerland; fdurmont@lallemand.com

**Keywords:** acceptability, medicine, sublingual, formulation, pediatric, children, polyvalent mechanical bacterial lysate, respiratory tract infections, prophylaxis, ClinSearch acceptability score test (CAST)

## Abstract

In pediatrics, acceptability has emerged as a key factor for compliance, and consequently for treatment safety and efficacy. Polyvalent mechanical bacterial lysate (PMBL) in 50-mg sublingual tablets is indicated in children and adults for the prophylaxis of recurrent respiratory tract infections. This medication may be prescribed in children over 3 years of age; the appropriateness of this sublingual formulation should thus be demonstrated amongst young children. Using a multivariate approach integrating the many aspects of acceptability, standardized observer reports were collected for medication intake over the course of treatment (days 1, 2, and 10) in 37 patients aged 3 to 5 years, and then analyzed in an intelligible model: the acceptability reference framework. According to this multidimensional model, 50-mg PMBL sublingual tablets were classified as “positively accepted” in children aged 3 to 5 years on all three days of evaluation. As the acceptability evaluation should be relative, we demonstrated that there was no significant difference between the acceptability of these sublingual tablets and a score reflecting the average acceptability of oral/buccal medicines in preschoolers. These results highlight that sublingual formulations could be appropriate for use in preschoolers.

## 1. Introduction

Respiratory tract infections (RTIs), which may affect the sinuses, throat, airways, or lungs, encompass several common human diseases such as the common cold, sinusitis, otitis media, bronchitis, or pneumonia. RTIs are particularly common in pediatrics, especially during the winter season. Polyvalent mechanical bacterial lysate (PMBL) is effective in adults and children for preventing RTI [1]. This bacterial lysate, which is obtained by mechanical lysis, is derived from 13 strains of inactivated pathogenic bacteria, including the most commonly occurring pathogens of the lower and upper respiratory tract (*Staphylococcus aureus*, *Streptococcus Viridans*, *Streptococcus pyogenes*, *Klebsiella pneumoniae*, *Klebsiella ozaenae*, *Haemophilus influenzae*, *Neisseria catarrhalis* and *Streptococcus pneumoniae*). Because bacterial lysates are mainly employed in the treatment of RTIs, we should start to consider the complexity of the innate response in the airway epithelium, particularly in the oropharyngeal region, which represents the first point of contact for inhaled foreign organisms. The protective arsenal of the upper airway epithelium is provided in the form of physical barriers and a vast array of receptors and antimicrobial compounds that constitute the innate immune system. Many of the known innate immune receptors, including Toll-like receptors and nucleotide oligomerization domain-like receptors, are expressed by the airway epithelium, leading to the production of proinflammatory cytokines and chemokines that affect microorganisms directly or by recruiting immune cells, such as neutrophils, dendritic cells, natural killer cells, and T cells, to the site of infection. Remarkably, in experimental animal models, the release of soluble factors, most likely anti-microbial peptides, by the airway epithelium seems to play a pivotal role in the protective effect exerted by bacterial lysates against respiratory infections [2]. Thus, if these innate mechanisms can be activated by the direct binding of pathogen-associated molecular patterns (PAMPs) to immune receptors present on mucosal cells, it can be easily envisaged that bacterial lysates with the same PAMPs can similarly trigger these physical mucosal barriers. Nevertheless, in order to achieve this effect, administration is supposed to be locoregional (for example sublingual or intranasal), as other routes of administration, including *per os*, would result in insufficient contact of bacterial lysates with oropharynx mucosal tissue, thus bypassing the activation of this relevant protection at the gate of entry of respiratory pathogens.

PMBL sublingual tablets (50 mg) are indicated in Europe for the prophylaxis of recurrent RTIs in adults and children from 5 years of age onwards. Recurrent RTIs can be diagnosed in children after excluding pathologies favorable to recurrent RTIs (e.g., cystic fibrosis, immune deficiencies), when at least one of the following conditions is met: at least six RTIs per year; at least one upper RTI monthly in the period from September to April (in Europe); and/or at least three lower RTIs per year [3]. In Poland, these tablets, which are intended to be dissolved under the tongue, are also indicated in children over 3 years of age. Taste, lack of cooperation, difficulties in co-ordination, and keeping medication at the site of absorption as well as risk of choking and aspiration could be some disadvantages of sublingual administration in young children [4]. In pediatrics, acceptability, defined as the overall ability and willingness of the patient to use and his/her caregiver to administer the medicine as intended, has emerged as a key factor for compliance and consequently, for treatment safety and effectiveness [5]. Therefore, the appropriateness of the formulation and pharmaceutical form of PMBP should be demonstrated among young children. Herein, we used a standardized assessment method, the ClinSearch Acceptability Score Test^®^ (CAST) [6,7], to investigate the acceptability of 50-mg PMBL sublingual tablets in children aged 3 to 5 years.

## 2. Materials and Methods

### 2.1. Study Design, Setting, and Regulatory Aspects

This monocentric, longitudinal, and strictly observational study was conducted in a medical center in Lublin (Poland) between July and August 2020.

According to local regulations the present study did not need ethical review and approval. The study protocol was reported to the Polish Competent Authority (URPL—Urzad Rejestracji Produktow Leczniczych, Wyrobow Medycznych i Produktow Biobojczych) for notification on 10 June 2020.

### 2.2. Participants and Sample Size

Patients were from 3 to 5 years of age, initiating a standard treatment (one tablet per day before eating for 10 consecutive days per month, for a total period of three consecutive months) of 50-mg PMBL sublingual tablets (Ismigen, Lallemand Pharma AG, Massagno, Switzerland), with verbal consent obtained from the parent(s)/legal representative. All eligible patients who met these inclusion criteria were approached by the investigators without any randomization processes. Participants were included in the study on a voluntary basis.

A minimum of 30 fully completed electronic case report forms (eCRFs) were required for analysis. This sample size was based on the primary endpoint of the study, for which at least 30 patients were necessary to assess acceptability with a satisfactory precision using the CAST methodology [6,7].

### 2.3. Data Collection

The parent(s)/legal representative observing the child taking the medication completed the eCRF, which was accessible on a web-based platform, for the first medicine intake (day 1, D1), and then for the medicine intake occurring 1 day later (day 2, D2) and 10 days later at the end of the first treatment period (day 10, D10).

The observers reported patient characteristics on D1: sex, age, and previous exposure to study treatment. For each medicine intake (D1, D2, and D10), data on several observable behaviors were collected as described henceforth. (1) The result of intake (whether the required dose was fully or partly taken, or not taken at all) was reported. Observers were required to check after 1 min if the tablet was still under the tongue. If not, we considered that the drug was not under the tongue long enough and consequently, “partly taken” was to be ticked. (2) The patient’s reaction using a three-point facial hedonic scale (positive, neutral, or negative) was recorded. (3) The time needed to prepare and to administer the required dose of medication was assessed and classified as short (1 min and less), medium (from 1 min to 2 min and 30 s), or long (longer than 2 min and 30 s). In addition, information on the following methods used to ease/achieve administration was reported, resulting in six binary variables with two possible values—used or not: (4) division of the intake of a dose which could not be taken as a whole; (5) alterations with regard to the intended form of use (modifying the dosage form or using another route/mode of administration), where observers were required to check after 30 s if the tablet was still under the tongue (if not, it was considered that the child had swallowed the sublingual tablet and consequently, “use of another route/mode of administration” was to be ticked); (6) use of food/drink to mask the drug taste; (7) use of a device not provided with the medication; (8) use of a reward; and (9) use of restraints (the patient had to be made to take the medication). For each method ticked the observers were requested to specify any further information in a text field. The observers had the opportunity to report text information related to any other unspecified method used.

Each evaluation of intake by each patient corresponded to a particular combination of an observed measure (e.g., fully taken) for each of the nine aforementioned observational variables (e.g., result of the intake) describing the many aspects of acceptability.

### 2.4. Recoding and Handling of Missing Values

Based on information in text fields and coding instructions (the observers had to check after 30 s and 1 min if the tablet was still under the tongue), some data for the nine observational variables could be recoded, using the worst case for acceptability scoring.

The R package missMDA [8] was used to handle missing values in multivariate data analysis for the evaluations of 50-mg PMBL sublingual tablets collected in this study. Imputation of the observed measures—categories—took into account both similarities between evaluations and relationships between the nine observational variables. The missMDA package provided a completed indicator matrix where the value can be considered as a degree of membership to the related category of each observational variable. Each missing value in the dataset was imputed with the most plausible category in multivariate data analysis.

### 2.5. Data Analysis

Acceptability scoring was performed using the acceptability reference framework: a three-dimensional map juxtaposing the positively and negatively accepted profiles. As described hereafter, multivariate analyses mined a large set of 1562 standardized evaluations of the intake of many oral/buccal medicines in children under 12 from seven countries (France, United Kingdom, Germany, Norway, Morocco, India and Japan) to summarize the main information into this intelligible tool.

First, a multiple correspondence analysis (MCA) summarized the variability between the evaluations (combinations of observed measures) and the key relationships between the observed measures themselves in a low-dimensional Euclidean space: the three-dimensional acceptability map. The three dimensions of the map revealed those associations and dissociations of observed measures that contributed the most to explaining variability observed in the data. The interpretation of the map is based on the distance between elements: proximities on the map express similarities. Observed measures that were close on the map were often selected together in the evaluations. Similarly, evaluations completed in a comparable manner converged on the map. Subsequently, a clustering process—hierarchical clustering on principal components and k-means consolidation—gathered the most similar evaluations (the closest on the map) into two coherent and meaningful clusters. The clusters that were described by the categories were significantly overrepresented in their subset of evaluations in comparison to a random distribution: v-test value greater than 1.96 (*p*-value < 0.05). All the “positive” observed measures naturally emerged in the first cluster, defining the “positively accepted” profile materialized by a green area on the map, while all the “negative” observed measures were over-represented in the second cluster, defining the “negatively accepted” profile materialized by a red area on the map.

The evaluations collected in this study were included in the multivariate analyses as supplementary information, with no influence on the factorial method. The evaluations of 50-mg PMBL sublingual tablets were thus plotted on the three-dimensional map, allowing for scoring process implementation.

The medicine under investigation was positioned at the barycenter of all the evaluations collected in this study. Confidence ellipses surrounding the barycenter for all dimension pairs defined an area containing its true position with 90% probability if the experiment was to be repeated. Each ellipse was made of 1000 points. Each point was assigned to one of the clusters. The proportion of points belonging to the different clusters was then recorded. If the barycenter, along with the entire confidence ellipsis surrounding it, belonged to the green area of the map, the medicine was classified as accepted. A minimum of 30 evaluations was required to obtain a reliable acceptability score.

Acceptability evaluation must necessarily be relative, and consequently the acceptability score of 50-mg PMBL sublingual tablets was compared to the average score of oral/buccal medicine acceptability in preschoolers. This acceptability score was reached using 424 evaluations of 160 distinct medicines taken by children aged 3 to 5 years from the dataset that gave rise to the acceptability reference framework (Table S1). Distinct acceptability scores are significantly different if confidence ellipses do not overlap on the map.

Subsequently, the evaluations of 50-mg PMBL sublingual tablets were partitioned into three subgroups according to the day of evaluations: D1, D2, and D10. Each “day” was positioned on the reference framework to study acceptability over time. Similarly, the influence of age, sex, and treatment exposure on acceptability was investigated.

The video abstract illustrates the mapping, clustering, and scoring processes described in this section.

For all the subgroups of interest that were compared, the significance of the differences observed in terms of patients’ characteristics (sex, age, and previous exposure) and observational variables for the acceptability scores was assessed. For each of those categorical variables, a test was used to determine whether there was a statistically significant difference between the observed frequencies and the expected frequencies in categories of a contingency table. When there was a minimum expectation of five for 80% of the categories of the contingency table without any null expectation, the Pearson’s chi-squared test was used; alternatively, Fisher’s exact test was used.

Data analyses were performed using R version 1.0.136^©^ (RStudio Team (2016). RStudio: Integrated Development for R. RStudio, Inc., Boston, MA, USA). The R package FactoMineR [9] was used to perform mapping and clustering processes.

## 3. Results

In this section we analyzed evaluations from 37 participants aged 3 to 5 years old using the acceptability reference framework to objectively establish the acceptability profile of 50-mg PMBL sublingual tablets in this age group.

### 3.1. Participants

Over the course of 18 days, 37 patients were included in the study (D1 completed), and only two patients were lost to follow-up 10 days later. Information on D1 and D2 was completed for one patient lost to follow-up, while this information was only completed for D1 for the other.

Table 1 presents the characteristics of the patients. Demographic data are missing for one patient.

### 3.2. Recoding and Handling of Missing Values

There were 43 recoded values impacting acceptability scoring (4.4% for the nine observational variables of the 108 evaluations). Some examples are as follows: the reported intake was recoded from “fully taken” to “partly taken” because the reported administration time was <60 s; the method “food/drink” was ticked based on the text-field “unspecified method”; and “use of another route/mode of administration” was ticked as the reported administration time was <30 s and the reported intake was “partly taken” (Table S2). In this last case, the medicine could have been swallowed or spat out prior to sufficient absorption taking place. As such, using the worst case for acceptability scoring, “use of another route/mode of administration”, was ticked due to the plausibility of swallowing having occurred.

There were 13 missing values (1.3%): two for the result of intake, four for the patient reaction, and seven for the preparation and administration times. Table S3 presents the completed indicator matrix resulting from the last step of the iterative MCA algorithm from the missMDA package. The completed dataset after imputation of the most plausible categories was used in multivariate data analysis.

### 3.3. Observer-Reported Outcomes

Table 2 presents the reported observed measures—without imputed missing values—describing acceptability for each day of evaluation (D1, D2, and D10).

No significant difference was observed for any of the variables between the three days of evaluation. It may nonetheless be noted that the percentage of negative reactions decreased from 30% on D1 to 17% on D2 and 6% on D10. At D10, 94% of reactions were reported as neutral or positive. The response “required dose not taken” was recorded for two patients on D1 and only one patient on D2 and D10. Each day, the majority of patients had taken the full required dose. As expected, the preparation and administration times were generally greater than 1 min as the medicine is intended to be placed and kept under the tongue until completely dissolved. Administration times lower than 1 min were thus related to a required dose that was partly or not taken.

Use of food/drink—either mixed with the drug or taken just before or after administration—was the most frequently reported method used to help with taking the medicine; the observer reported that the patient drank water after intake in most of these cases. Only one explicitly specified that this was due to the drug taste. Use of hot chocolate, fruit juice, and candy was also reported. The tablet was dissolved prior administration for two patients and broken into pieces for three others. Alteration of the intended use due to difficulty with sublingual administration was specified for only one patient on D1 who kept the “majority on the tongue”, while the medicine was swallowed after a time in the mouth for two other patients. The remaining alterations were due to re-coding. Use of a reward was the third most-reported method used to ease/achieve administration, e.g., with sweets or toys. For one patient a teaspoon was used to help with taking the medicine. The intake of the required dose was divided for two patients. Finally, only one patient had to be made to take the medicine on D1, and one other on D10.

Each evaluation corresponds to a combination of an observed measure for each of the nine observational variables. The following combination reflecting an intended medicine use without problems was the most used (13% of the 108 evaluations): “fully taken”, “positive reaction”, “medium time”, “no divided dose”, “no food drink”, “no alteration”, “no extra device”, “no reward”, and “no restraint”. The same combination with the category “long time” replacing “medium time” was the second most common (9%), and the combination with the categories “long time” and “neutral reaction” replacing “medium time” and “positive reaction” respectively was the third (6%) most observed.

### 3.4. Acceptability Scoring

**Figure 1:**
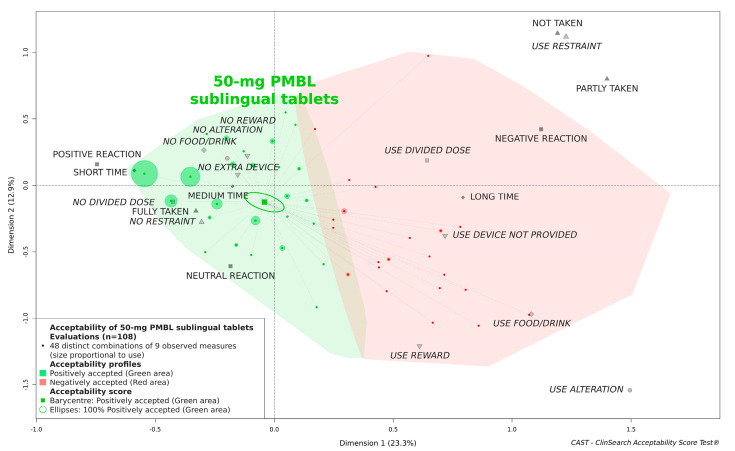
Acceptability of 50-mg polyvalent mechanical bacterial lysate (PMBL) sublingual tablets.

According to the acceptability reference framework, the 50-mg PMBL sublingual tablets were classified as “positively accepted” in children aged 3 to 5 years (Figure 1). The barycenter of all the evaluations collected in this study (37 on D1, 36 on D2, and 35 on D10), along with the confidence ellipses surrounding them, was fully located in the green area of the acceptability map.

**Figure 2:**
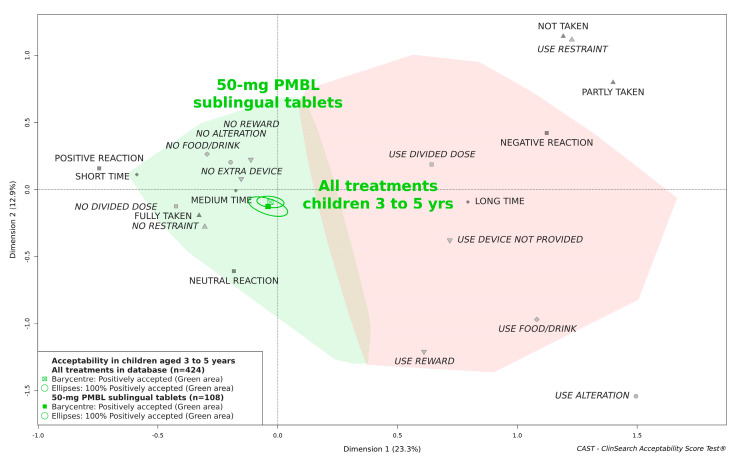
Acceptability of 50-mg PMBL sublingual tablets compared with a large scale of treatments in children aged 3 to 5 years.

Figure 2 shows the overlapping confidence ellipses demonstrating that there was no significant difference between the overall score of 50-mg PMBL sublingual tablets and the score which reflected the average acceptability of all oral/buccal medicines tested in preschoolers.

**Figure 3:**
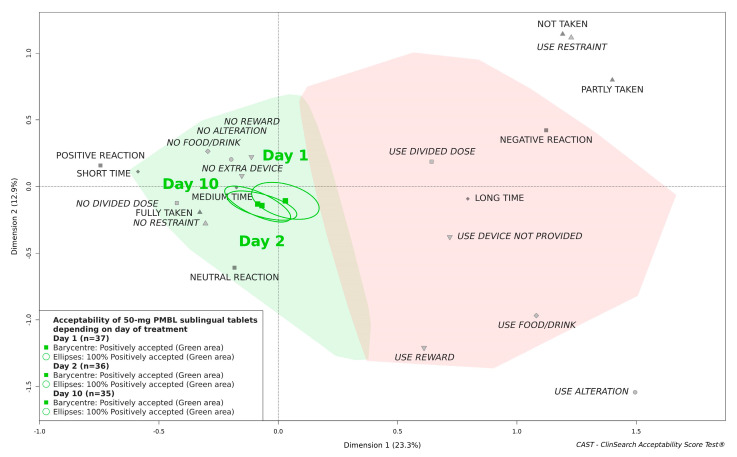
Acceptability of 50-mg PMBL depending on the day of treatment.

Although a slight acceptability improvement was observed over time—D2 and D10 were located further from the negative area materialized in red on the right of the map than D1—the 50-mg PMBL sublingual tablets were scored as “positively accepted” on all days of the evaluation (Figure 3).

We investigated in secondary analyses the influence of patient characteristics on the acceptability of 50-mg PMBL sublingual tablets.

**Figure 4:**
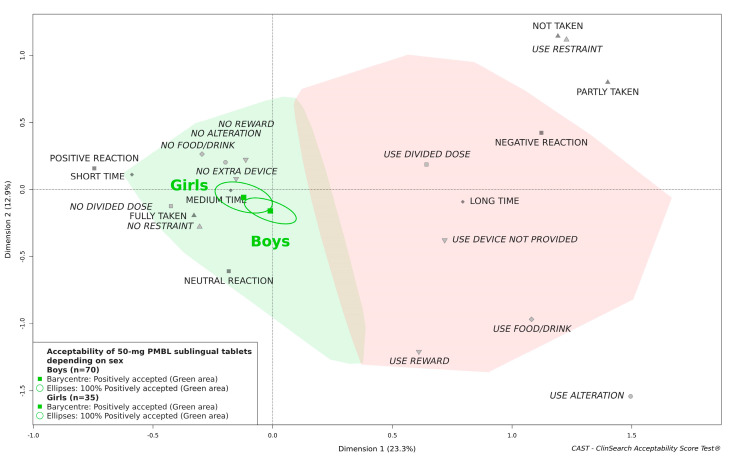
Influence of sex on the acceptability of 50-mg PMBL sublingual tablets.

**Figure 5:**
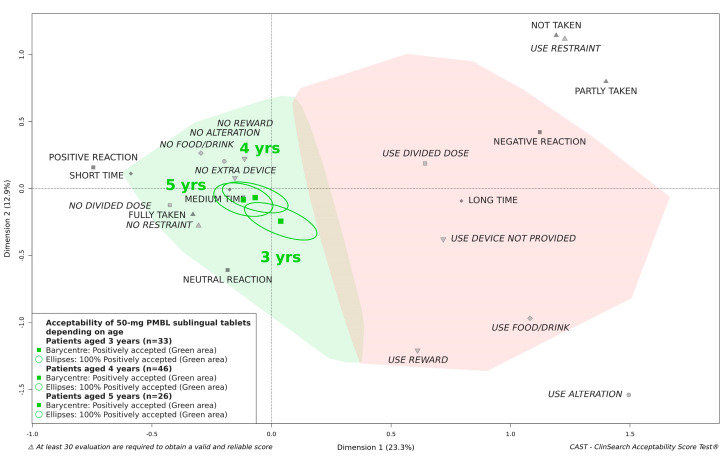
Influence of age on the acceptability of 50-mg PMBL sublingual tablets.

**Figure 6:**
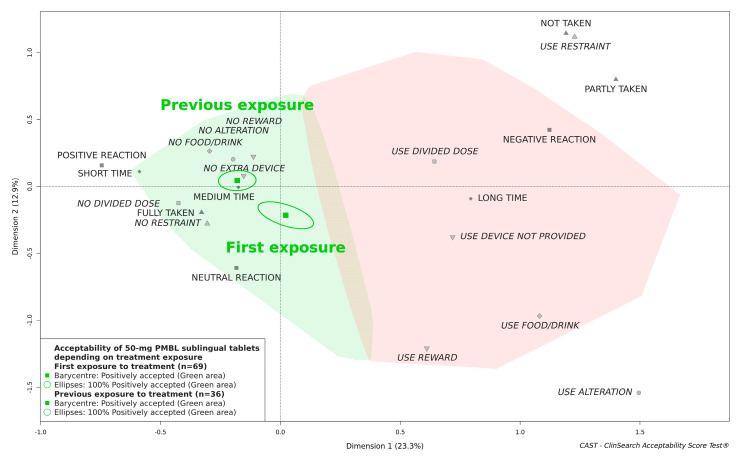
Influence of treatment exposure on the acceptability of 50-mg PMBL sublingual tablets.

Figure 4 illustrates there was no significant difference in acceptability between boys and girls. The 50-mg PMBL sublingual tablets in children aged 4 and 5 years did appear to be located further from the negative area than in children aged 3 years, but the medicine was classified as “positively accepted” in all age groups (Figure 5). Likewise, the acceptability score was significantly better in children who had been previously treated with the medicine, but the medicine remained accepted in children who undertook their first treatment period (Figure 6).

There was no significant difference between patients aged 3, 4, or 5 years old in terms of sex (Fisher’s exact test, *p* = 0.9) and treatment exposure (Fisher’s exact test, *p* = 0.25). Similarly, there was no significant difference between boys and girls regarding treatment exposure (Pearson’s chi-squared test, *p* = 0.71). Thus, there was no underlying confounding factor related to the acceptability differences highlighted.

## 4. Discussion

The acceptability reference framework allowed for the simultaneous consideration of the different combinations of observed measures reflecting the distinct behaviors of 50-mg PMBL sublingual tablet users in real-life conditions. According to this tool, 50-mg PMBL sublingual tablets were considered as accepted in children aged from 3 to 5 years old: the medicine is fully located in the green zone of the model defining the “positively accepted” profile. In the absence of a gold standard, classifications cannot be absolute and comparative applications of the tool must be used. Therefore, the acceptability score of 50-mg PMBL sublingual tablets was compared with the average medicine acceptability in preschoolers within the acceptability reference framework. The latter score was based on many evaluations of a wide range of oral/buccal medicines in children aged from 3 to 5 from seven countries with various cultures. These 424 evaluations are a part of the dataset that gave rise to the acceptability reference framework. There was no significant difference between the two acceptability scores.

The 50-mg PMBL sublingual tablets appeared to be “positively accepted” regardless of the day of evaluation. The medicine was indeed positioned on the green zone of the acceptability reference framework on D1, D2, and D10. Furthermore, there was no significant difference between these three scores and the average medicine acceptability score in preschoolers. As the medicine was accepted from the first day of the treatment period, patient training appears to be non-essential in children aged from 3 to 5 years. In addition, although acceptability appeared to be significantly better in children who had been previously treated with the medicine, the medicine was also accepted in children in their first treatment period. Supporting good acceptability over time, these findings argue in favor of positive acceptability for the successive treatment periods as the standard treatment is one 50-mg tablet per day for 10 consecutive days per month, for a total period of three consecutive months.

These results highlight the positive acceptability of 50-mg PMBL sublingual tablets over time in real-life conditions, establishing this formulation and pharmaceutical form as appropriate in children from 3 to 5 years of age. A previous study investigating compliance to sublingual PMBL treatment in 85 children aged 10 months to 10 years highlighted good compliance in children up to 5 years using a slightly modified conventional dummy as a device to facilitate the administration of the crushed tablet [10]. This controlled randomized clinical trial established the efficacy and tolerability of PMBL treatment on a clinical level. Considering the satisfactory acceptability in preschoolers, such a simple handmade device which could be perceived as a limitation of the trial does not seem necessary to ensure acceptability and, consequently, compliance and prophylaxis efficacy in these young patients.

Beyond RTIs, sublingual formulations should be of interest to provide suitable alternatives to solid oral dosage forms in preschoolers given the ongoing need for age-appropriate formulations in pediatrics, especially in younger children [11].

## Figures and Tables

**Table 1:** Characteristics of the 37 patients.

Characteristics	*n* (%)
**Sex**	
Girl	12 (33)
Boy	24 (67)
Missing data	1
**Age (years)**	
3	11 (31)
4	16 (44)
5	5 (25)
Missing data	1
**Treatment exposure**	
Previous exposure	12 (33)
First exposure	24 (67)
Missing data	1

**Table 2:** Observer-reported outcomes for each day of evaluation.

Outcomes	Day 1 (*n* = 37)	Day 2 (*n* = 36)	Day 10 (*n* = 35)	Statistical Test
**Result intake**				
Fully taken	21 (57) ^1^	24 (67)	19 (58)	F ^2^: *p* = 0.88
Partly taken	14 (38)	11 (30)	13 (39)	
Not taken	2 (5)	1 (3)	1 (3)	
Missing data			2	
**Patient reaction**				
Positive reaction	14 (38)	15 (43)	15 (47)	χ^2 3^: *p* = 0.17
Neutral reaction	12 (32)	14 (40)	15 (47)	
Negative reaction	11 (30)	6 (17)	2 (6)	
Missing data		1	3	
**Preparation and administration time**				
Short time	5 (14)	5 (15)	4 (12)	χ^2^: *p* = 0.54
Medium time	15 (43)	11 (33)	18 (55)	
Long time	15 (43)	17 (52)	11 (33)	
Missing data	2	3	2	
**Divided dose**				
No divided dose	35 (95)	36 (100)	34 (97)	F: *p* = 0.65
Use of divided doses	2 (5)	0 (0)	1 (3)	
**Food/drink** ^4^				
No food/drink	26 (70)	24 (67)	26 (74)	χ^2^: *p* = 0.78
Use of food/drink	11 (30)	12 (33)	9 (26)	
**Alteration** ^5^				
No alteration	27 (73)	29 (81)	27 (77)	F: *p* = 0.74
Use of alteration	10 (27)	7 (19)	8 (23)	
**Extra device**				
No extra device	36 (97)	35 (97)	34 (97)	F: *p* = 1
Use device not provided	1 (3)	1 (3)	1 (3)	
**Reward**				
No reward	32 (86.5)	32 (89)	30 (86)	χ^2^: *p* = 0.92
Use of reward	5 (13.5)	4 (11)	5 (14)	
**Restraint**				
No restraint	36 (97)	36 (100)	34 (97)	F: *p* = 0.77
Use of restraint	1 (3)	0 (0)	1 (3)	

^1^*n*(%): number and percentages; ^2^ F: Fisher’s exact test; ^3^ χ^2^: Pearson’s chi-squared test; ^4^ either mixed with the drug or taken just before or after administration; ^5^ either modification of dosage form prior administration or use another route/mode of administration.

## Data Availability

Data underlying the study cannot be made publicly available due to legal and ethical considerations. European Union (GDPR) laws restrict the public sharing of personally identifiable data. Requests for data will be processed according to the French MR-003 Code of conduct by the data controller, ClinSearch, which allows for the use of data for the purpose of reproducing study results. Requests to access the data for this purpose may be sent to the data protection officer of Lallemand Pharma AG: officelp@lallemand.com, and researchers outside the European Union will need to sign a transfer agreement.

## Supplementary Materials

The following are available online at https://www.mdpi.com/1999-4923/13/2/294/s1. Table S1. Patient and medicine characteristics of the 424 evaluations in children from 3 to 5 years of age within the dataset that gave rise to the acceptability reference framework; Table S2. Recoding based on coding instructions and information in text fields; Table S3. Completed indicator matrix for variables with missing values.
