# Supplementary material for: Acceptability of a Sublingual Drug Formulation for Respiratory Tract Infections in Children Aged 3 to 5 Years
Source: Pharmaceutics. 2021 Feb 23;13(2):294. doi: 10.3390/pharmaceutics13020294 (PMC7926363; doi:10.3390/pharmaceutics13020294)
Supplement: Supplementary file 1 [file pharmaceutics-13-00294-s001.pdf]

pharmaceutics

Supplementary Materials: Acceptability of a Sublingual Drug Formulation for Respiratory Tract Infections in Children Aged 3 to 5 Years

Andrzej Emeryk, Thibault Vallet, Ewelina Wawryk-Gawda, Arkadiusz Jędrzejewski, Frederic Durmont and Fabrice Ruiz

Table S1. Patient and medicine characteristics of the 424 evaluations in children from 3 to 5 years of age within the dataset that gave rise to the acceptability reference framework.

Patients and medicines characteristics	n(%)
**Sex of patients**	
Girl	176 (42)
Boy	246 (58)
missing data	2
**Age of patient (years)**	
3	186 (43)
4	121 (29)
5	117 (28)
**Treatment exposure**	
Previous exposure	213 (50)
First exposure	211 (50)
**Type of care**	
Home	87 (21)
Hospital	337 (79)
**Country**	
Morocco	168 (40)
France	100 (24)
India	78 (18)
Japan	34 (8)
Norway	27 (6)
Germany	11 (3)
England	6 (1)
**Pharmaceutical products**	
Doliprane 2.4perc. Oral suspension sugar free	29 (7)
Cefaclor 10perc. Fine granules	21 (5)
Depakine 57.64mg-1ml. Syrup	18 (4)
Oroken children 100mg-5ml. Powder for oral suspension	17 (4)
Orapred 20mg. Effervescent tablet	13 (3)
Celestene 0.05perc. Drops for oral solution	13 (3)
Aerius 0.5mg-1ml. Oral solution	13 (3)
Fucidine 250mg-5ml. Oral suspension	11 (3)
Cotipred 20mg. Effervescent tablet	11 (3)
Sintrom 4mg. Tablet	10 (2)
Josacine 125mg-5ml. Granules for oral suspension	9 (2)
Doliprane 300mg. Powder for oral solution	9 (2)
Crocin ds 240mg. Syrup	9 (2)
Other (<2%)	241 (57)

**5th level (chemical substance) of the Anatomical Therapeutic Chemical (ATC) classification system**	
Paracetamol	63 (15)
Prednisolone	35 (8)
Valproic acid	26 (6)
Cefaclor	21 (5)
Co-amoxiclav	19 (5)
Cefixime	17 (4)
Desloratadine	15 (4)
Josamycin	13 (3)
Fusidic acid	13 (3)
Betamethasone	13 (3)
Acenocoumarol	10 (2)
Amoxicillin	10 (2)
Phenobarbital	9 (2)
Other (<2%)	158 (38)
missing data	2
**2nd level (therapeutic subgroup) of the ATC classification system**	
Antibacterials	122 (29)
Analgesics	65 (15)
Antiepileptics	55 (13)
Corticosteroids	51 (12)
Antihistamines	21 (5)
Antivirals	12 (3)
Psycholeptics	11 (3)
Antithrombotic agents	11 (3)
Constipation	10 (2)
Cough and cold preparations	8 (2)
Other (<2%)	58 (13)
**Dosage forms**	
Syrup	77 (18)
Oral suspension	57 (13)
Powder for oral suspension	45 (11)
Tablet	38 (9)
Oral solution	34 (8)
Effervescent tablet	28 (7)
Fine granules	24 (6)
Powder for oral solution	23 (5)
Granules for oral suspension	17 (4)
Drops for oral solution	17 (4)
Coated tablet	16 (4)
Divisible tablet	12 (3)
Orally disintegrating tablet	8 (2)
Other (<2%)	28 (6)
**Dosage form categories**	
Oral liquid preparations	339 (80)
Solid oral dosage form	74 (17)
Buccal preparations	11 (3)

Table S2. Recoding based on coding instructions and information in text fields.

Recoding	Patient (Evaluation Per Day)

As the reported intake was 'fully taken' although the reported administration time was < 60 sec, the result of intake has been re-coded from 'fully taken' to 'partly taken'	4 patients (2 at D1, 2 at D2, 4 at D10)
As 'use another route/mode of administration' was not ticked although the reported administration time was < 30 sec and the reported intake was different from 'not taken', 'use another route/mode of administration' has been ticked	10 patients (6 at D1, 4 at D2, 5 at D10)
As 'use another route/mode of administration' was ticked, the result of intake has been re-coded from 'fully taken' to 'partly taken'	1 patient (1 at D1, 1 at D2, 1 at D10)
Based on text-field of 'divided dose' (hereafter), 'modification of dosage form' has been ticked	
"split in two"	1 patient (1 at D10)
"in two pieces"	1 patient (1 at D1)
Based on text-field of 'divided dose' (hereafter), 'divided dose' has been unticked and 'use another route/mode of administration' ticked	
"30 seconds, the tablet was under the tongue, then crushed in the mouth and swallowed"	1 patient (1 at D1)
Based on text-field of 'mix food/drink' (hereafter), 'modification of dosage form' has been ticked	
"100 ml of hot chocolate"	1 patient (1 at D1, 1 at D2, 1 at D10)
Based on text-field of 'mix food/drink' (hereafter), 'use device not provided' has been ticked	
"a teaspoon of water"	1 patient (1 at D1, 1 at D2, 1 at D10)
Based on text-field of unspecified method (hereafter), 'take food/drink' has been ticked	
"drank water afterwards"	1 patient (1 at D1)
"drank water after dispersion"	1 patient (1 at D2, 1 at D10)
"a mouthful of water, 1.5 minutes after intake"	1 patient (1 at D2)
"after orodispersion of the tablet, the child wanted to drink water"	1 patient (1 at D1)
"needed to drink water due to poor taste"	1 patient (1 at D1)
"drank water after dispersion and swallowing of tablet"	1 patient (1 at D2)
"drank water after having swallowed"	1 patient (1 at D10)

1

Table S3. Completed indicator matrix for variables with missing values.

**Patient**	**Day**	**Result Intake**			**Patient Reaction**			**Preparation and Administration Time**		
		Fully taken	Partly taken	Not taken	Positive	Neutral	Negative	Short	Medium	Long
A	2	1	0	0	0	1	0	−0.087	**0.654**	0.433
B	10	1	0	0	0.378	**0.486**	0.135	0	1	0
C	10	0.4	**0.599**	0.001	1	0	0	1	0	0
D	2	1	0	0	0	1	0	−0.087	**0.654**	0.433
	10	0	1	0	1	0	0	0.268	**0.468**	0.264
E	1	0	0	1	0	0	1	0.526	**0.705**	−0.23
F	10	**0.844**	0.111	0.045	0	1	0	0	0	1
G	10	0	1	0	0.356	**0.425**	0.219	**0.438**	0.229	0.333
H	2	1	0	0	1	0	0	0.044	0.49	0.466
	10	1	0	0	**0.616**	0.234	0.15	0	0	1
I	2	1	0	0	**0.432**	0.352	0.216	0	0	1
J	1	0	1	0	0	0	1	**0.602**	0.21	0.189

bold value: the most plausible category.
